# Malignant Serous Effusions among Hospital In-patients in a Tertiary Care Hospital: A Descriptive Cross-sectional Study

**DOI:** 10.31729/jnma.7145

**Published:** 2022-02-28

**Authors:** Ramesh Dhakhwa, Shreya Sapkota, Anju Maharjan, Sailesh Pradhan

**Affiliations:** 1 Department of Pathology, Kathmandu Medical College and Teaching Hospital, Sinamangal, Kathmandu, Nepal

**Keywords:** *cytodiagnosis*, *histology*, *malignant*, *serous effusions*

## Abstract

**Introduction:**

Cell block technique is an adjunct to conventional smears in the diagnosis of malignancy in effusion fluid. It aims at retrieving cellular material and concentrating cells in a small field, with preservation of cytomorphologic details. The objective of this study was to find the proportion of malignant serous effusions using cell block technique among hospital in-patients in a tertiary care centre.

**Methods:**

This was a descriptive cross-sectional study conducted among patients visiting a tertiary care centre between 1^st^ June 2020 to 30^th^ November 2020. Ethical approval was taken from the Institutional Review Committee (Reference number: 305202001). Using a convenience sampling method, 96 hospital in-patients were included in the study. Serous effusions were evaluated by conventional smears and cell block sections. Data was analysed using the Statistical Package for the Social Sciences version 23. Point estimate at 95% Confidence Interval was calculated along with frequency, percentage, mean and standard deviation.

**Results:**

Among 96 hospital in-patients, 15 (15.62%) (8.35-22.88 at 95% Confidence Interval) were diagnosed as positive for malignancy by using cell block technique. By conventional smears, 80 (83.33%) cases turned out to be negative for malignancy, 13 (13.54%) were positive for malignancy and three (3.12%) were suspicious for malignancy. Of the three (3.12%) cases suspected for malignancy, two turned out to be positive for malignancy and one was found to be negative for malignancy on cell block technology.

**Conclusions:**

The proportion of malignant serous effusions was similar in comparison to other studies. Cell block technique could be routinely incorporated along with conventional smears for a more accurate diagnosis of malignancy on serous effusion.

## INTRODUCTION

Cytological assessment of serous fluids is an important investigation for diagnosis, staging, prognosis and management of malignancy as well as diagnosis of various non-neoplastic lesions.^1^ Cytological examination using conventional smearing is a simple, easy and inexpensive method but at times cytologists are prone to diagnostic pitfalls owing to paucity of representative cells and less clear cytomorphological features on conventional smears.^2-4^

Cell block technique (CBT) has now become widely accepted and recommended by many experts as an adjunct of conventional smears in the cytodiagnosis of effusion cytology.^3^ It retrieves cellular material and concentrates cells in a small field, with preservation of cytomorphologic details, and better demonstration of architectural patterns-such as acini, cell balls, papillae and rosettes-in a biopsy like fashion. It provides archived material that can be used for special stains and immunohistochemistry.^5-9^

The objective of this study was to find the proportion of malignant serous effusions among hospital in-patients of a tertiary care centre.

## METHODS

This was a descriptive cross sectional study conducted among hospital in-patients of Kathmandu Medical College Teaching Hospital (KMCTH), Sinamangal whose pleural and peritoneal fluids were submitted to the department of pathology for cytological evaluation between 1^st^ June 2020 to 30^th^ November 2020. The ethical approval was given by the Institutional Review Committee (Reference number: 305202001) of KMCTH prior to the commencement of the study. Consecutive sampling technique was used. The sample size for the study was calculated using the given formula:

n = Z^2^ × p × q / e^2^

  = (1.96)^2^ × 0.5 × (1-0.5) / (0.1)^2^

  = 96

Where,

n = minimum required sample size
Z = 1.96 at 95% Confidence Interval (CI)
p = prevalence taken as 50% for maximum sample size
q = 1-p
e = margin of error, 10%

We have included 96 samples of serous effusion. Already diagnosed cases of malignancy, recurrent effusions, and pericardial effusions were excluded from our study. Relevant demographic data were obtained from the requisition form provided with the specimens.

Each submitted fluid sample was divided into two equal portions. The first portion was subjected to conventional smear cytology and the second portion was utilised for cell block technique, using 5ml of 10% formalin as a fixative. After one hour, the fluid was centrifuged at 2500rpm for 15 minutes. The supernatant was discarded and 3ml fresh 10% formalin was added to the sediment and kept at room temperature for one day. The sediment containing the cell button of the fluid sample was scooped out into a filter paper and processed into formalin fixed paraffin embedded blocks, from which 4-6 micron thick sections were cut and stained with H and E stain. Both conventional smears and cell block sections were evaluated for cellularity, architectural pattern, cell arrangement and cytomorphologic features. Each case was reported either to be negative for malignancy, positive for malignancy or suspicious for malignancy by both techniques. Provisional diagnoses made on conventional smears were again evaluated and revised after examination of cell block slides.

The data was entered and analysed using the Statistical Package for the Social Science (SPSS version 23.0). Point estimate at 95% Confidence Interval was calculated along with frequency, percentage for binary data and mean, standard deviation for continuous data.

## RESULTS

Out of the 96 hospital in-patients with serous effusion, 15 (15.62%) (8.35-22.88 at 95% Confidence Interval) were diagnosed as positive for malignancy by using cell block technique (Table 1). Of the three (3.12%) cases which were suspicious of malignancy on conventional smear, two were positive for malignancy and one was negative for malignancy on cell block preparation. All cases of malignancy were metastatic adenocarcinoma, however the primary site of tumour could not be determined based on cytology alone. Out of 96 cases, 80 (83.33%) were diagnosed as negative for malignancy and 13 (13.54%) positive for malignancy on conventional smears whereas three (3.12%) were suspicious of malignancy.

**Table 1:** Diagnosis by using cell block technique (n = 96).

Interpretation	n (%)
Positive	15 (15.62)
Negative	81 (84.48)

**Figure 1:**
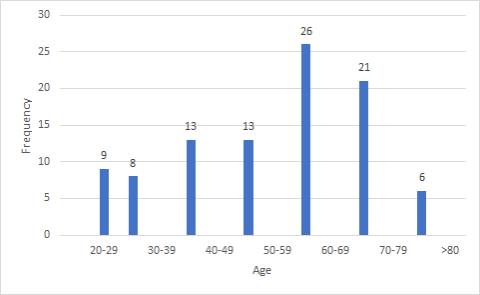
The age of the patients in range from which samples were taken (n= 96).

**Figure 2:**
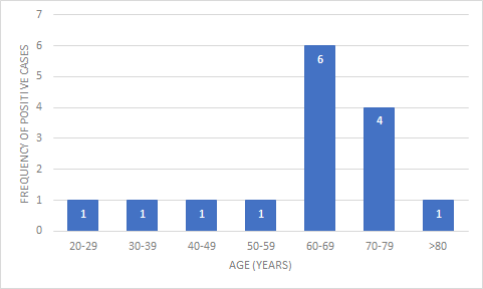
Age wise distribution of positive cases for malignancy on cell block technique (n=15).

There was a predominance of pleural fluid 62 (65%) followed by peritoneal fluid 34 (35%). Male patients 61 (63.55%) outnumbered female patients 35 (36.45%). Malignant effusion was slightly more common in male (male:female ratio being 1.3:1). The age of the patients ranged from 20 to 84 years with a mean of 65 years (Figure 1). Maximum number of samples was from the 60-69 years age group 26 (27.08%). Also the number of malignancy in this age group was the highest 6 (23.07%) (Figure 2).

## DISCUSSION

Cytological examination of body fluids commonly involves the use of direct or sediment smears (conventional smear technique).^7^ It is considered an easy, simple and inexpensive method, which is used as a definitive test to guide patients' management. Although most cases can be diagnosed morphologically based on conventional smears, there are a number of cases in which an unequivocal diagnosis cannot be reliably established causing diagnostic dilemma.^3,8^ Due to cellular overlapping, delaying artefact, suboptimal processing and preparatory cytotechnique, there is lower diagnostic yield in conventional smear method. This residual material after smears are prepared is useful in increasing diagnostic yield by cell block method.^9^ Cell block technique is extremely useful in improving cell yield of thin serous effusions and ensure high diagnostic efficacy.^10^ This technique concentrates the retrieved material in a small field which appear as mini biopsies and are useful for diagnosis, pattern recognition, sub classification and identification of features that may otherwise be difficult to appreciate on non-cell block cytology preparation.^11^ Moreover, it provides archived material that can be used for special stains, immunohistochemistry, ultrastructure studies and molecular tests including cytogenetic and polymerase chain reaction based techniques.^12,13^ This might allow patients to benefit from targeted therapy without the need for additional invasive tissue sampling.^14^ We carried out a study to determine the utility of CBT in diagnosing malignancy in serous effusion in a tertiary care centre.

In our study, pleural fluid was the commonest serous effusion (65%) followed by peritoneal fluid (35%). Similar studies conducted by Thapar M, et al. and Saha R, et al. also found pleural effusion to be the most common effusion followed by peritoneal effusion.^6,15^ In the present study most common age group of serous effusion was 60 to 69 years. Malignant effusion was also the highest in this age group. The increased number of cases in this age group could be due to increased incidence of malignancy in the elderly

Out of the 15 positive cases of serous effusion on cell blocks, nine were malignant pleural effusion (60%) and six were peritoneal effusion (40%). Malignant pleural effusion was more common in male (6 out of 9) than in females whereas malignant peritoneal effusion was more common in females (4 out of 6). Based on cytology alone we could not determine the primary site of malignancy. However, there was evidence of primary lung carcinoma in four cases of malignant pleural effusion. Similarly among the six cases of malignant peritoneal effusion, we could trace history of primary ovarian carcinoma in two cases and gastrointestinal carcinoma in another two cases. In the remaining two cases, there was no known primary site of tumour. Datta et al found a higher number of malignant peritoneal effusion in females and higher number of malignant pleural effusion in males owing to the different incidences of primary site of tumour in their studies.^2,15^ Due to small number of cases in the present study we can not make a definitive statement regarding an increased incidence of malignant pleural effusion in male and increased incidence of peritoneal effusion in female.

When we evaluated the findings of conventional smear with cell block technique there was not much difference regarding the frequency of negative cases; 80 cases of negative effusion on conventional smear to 81 cases on CBT. One case was diagnosed as suspicious of malignancy on conventional smear due to presence of moderately pleomorphic clusters of cells. But when cell block preparation was examined those pleomorphic cells proved to be reactive mesothelial cells hence a confident diagnosis of negative for malignancy was possible on CBT. Similarly two other cases of suspicion of malignancy on conventional smears were confidently diagnosed as positive for malignancy on CBT. In these two cases cellularity was low on conventional smears and there was confusion whether the atypical cells were reactive mesothelial or malignant cells. However when cell block smears were examined neoplastic glands and signet ring cells could be appreciated and a confident diagnosis of positive for malignancy was given.

Khan N, et al. found similar results and concluded that cell blocks are particularly useful when the cytological abnormalities are misleading as in reactive mesothelial cells or in well differentiated adenocarcinoma. CBT demonstrates cytological architecture in a biopsylike fashion and multiple sections of the same material can be obtained for special stains and immunohistochemistry.^16^ Similar conclusions were drawn by Ford AG, et al. who conducted cellular studies of effusion by using smears and cell blocks.^17^ Shen SC, et al. reported pathognomic features of malignancy are better seen in cell blocks than in smears.^18^ Present study illustrates the usefulness of cell block technique in detecting malignant effusion when used with conventional smears.

However, there were limitations of the study as it included only pleural and peritoneal effusion and only a limited number of cases of malignant effusion could be included during the study period. Besides, application of immunohistochemical stains on cell block smears could have further highlighted the usefulness of CBT in determining the primary site of malignancy.

This was a single centre study with small sample size so we recommend future studies with larger sample size. The confirmatory diagnosis by using tissue biopsy and immunohistochemistry and other clinical findings were not included in our study.

## CONCLUSIONS

The proportion of malignant serous effusions in hospital in-patients was similar as noted in other studies. Cell block technique is a simple and easy technique and demonstrates better morphologic details and architectural pattern. It could be used for accurate diagnosis in suspicious cases of malignancy.
